# The Clinical Impact of Cardiovascular Thrombosis on Overall Survival in Patients With Hepatocellular Carcinoma After Transarterial Chemoembolization

**DOI:** 10.1002/cam4.71594

**Published:** 2026-02-08

**Authors:** Koji Fujita, Kei Takuma, Mai Nakahara, Hironobu Suto, Asahiro Morishita, Takashi Himoto, Keiichi Okano, Hideki Kobara

**Affiliations:** ^1^ Department of Gastroenterology and Neurology, Faculty of Medicine Kagawa University Takamatsu Japan; ^2^ Department of Gastroenterological Surgery, Faculty of Medicine Kagawa University Takamatsu Japan; ^3^ Department of Clinical Laboratory Medicine Kagawa Prefectural University of Health Sciences Takamatsu Japan

**Keywords:** antithrombotic agents, coronary artery diseases, liver cancer, stroke, transcatheter arterial chemoembolization

## Abstract

**Objectives:**

Progression of hepatocellular carcinoma (HCC) and cardiovascular thrombosis (CVT) has a bidirectional causal relationship. CVT complications will increase in patients with HCC due to etiology shift from viral hepatitis to metabolic dysfunction‐related steatohepatitis.

**Aim:**

This study aimed to evaluate the clinical impact of CVT, focusing on patients with HCC treated after transarterial chemoembolization.

**Methods:**

A retrospective cohort study enrolled 402 patients including 79 patients with CVT in a single university hospital. Cox proportional hazard model analysis was performed to identify independent prognostic factors. After adjusting for baseline characteristics by propensity score matching, the survival impact of the CVT complication was evaluated using the Kaplan–Meier curve.

**Results:**

A multivariate analysis determined that CVT complication was an independent risk factor for overall deaths in patients with HCC (HR = 1.751, IQR 1.203–2.548, *p* < 0.05). Propensity score matching generated a pair of 54‐patient cohorts. The median survival time of patients with CVT (1106 days) shortened to half compared to those without CVT (2707 days, HR = 2.298, IQR: 1.399–4.169, *p* = 0.0020). While recurrence‐free survival was not significantly different (*p* > 0.05), post‐recurrence survival was shorter in patients with CVT (2150 days vs. 1008 days, HR = 1.945, IQR: 1.150–3.740, *p* = 0.0188).

**Conclusions:**

Assuming that the expected life expectancy is only half that of uncomplicated cases of CVT, CVT might be a major prognostic factor in patients with HCC, following tumor burden and functional hepatic reserve.

**Abbreviations**
95% CI: 95% confidence interval
AIH: autoimmune hepatitis
ALBI score: albumin bilirubin score
CVT: cardiovascular thrombosis
DEB‐TACE: drug‐eluting beads TACE
HAIC: hepatic arterial infusion chemotherapy
HBV: hepatitis B virus
HCC: hepatocellular carcinoma
HCV: hepatitis C virus
ICG‐R15: indocyanine green retention rate at 15 min test
IQR: interquartile range
NASH: nonalcoholic steatohepatitis
PBC: primary biliary cholangitis
TACE: transarterial chemoembolization
TAE: transcatheter arterial embolization
TAI: transcatheter arterial infusion

## 1 Introduction

Hepatocellular carcinoma (HCC) is the sixth most common cancer worldwide with an increasing incidence [1]. Patients with HCC present a significant risk of venous thrombotic embolism because cancer and liver cirrhosis can perturb the hemostatic balance towards a prothrombotic state [2].

In recent years, the incidence of HCC has shifted from patients with virus‐related liver diseases to those with non‐viral etiologies, including alcohol‐ and metabolic dysfunction‐related fatty liver disease (MAFLD) [3]. MAFLD is a risk factor for arterial thrombosis, which is a major cause of death in patients with MAFLD [4].

In addition to platelet thrombosis, coagulation thrombosis, including deep venous thrombosis, frequently complicates cancer development [5]. Interaction with coagulation thrombosis may promote tumor growth and seeding. Venous thrombosis is the leading cause of death in patients with cancer [6].

Thus, cardiovascular thrombosis (CVT) bidirectionally correlates with the health status of patients with hepatocellular carcinoma. However, the clinical impact of CVT on patients with HCC has not been fully assessed. This study aimed to clarify the survival impact of CVT in patients with HCC, focusing on those treated with transarterial chemoembolization (TACE).

## 2 Materials and Methods

### 2.1 Ethics

This study was conducted in accordance with the ethical principles of the Declaration of Helsinki [7] after approval by the Institutional Review Board of our university, Faculty of Medicine (serial number: 2024‐178). We provided an opt‐out method for patients and their relatives by publishing a summary of this study on our university website [8].

### 2.2 Study Design

This retrospective observational study was conducted at a single university hospital and targeted patients with HCC treated with TACE. The primary endpoint was overall mortality. Liver‐related deaths were defined as deaths caused by cirrhosis or HCC. The date of recurrence was defined as the day when treatments were performed for the recurrent tumor, or the day when any treatments for the recurrent lesion were halted, thereafter excluding supportive care. The sample size was limited due to the retrospective nature of the study. To control for potential bias in the original cohort, propensity score matching was applied [9]. The study was performed in accordance with the STROBE statement [10].

### 2.3 Patients

**FIGURE 1:**
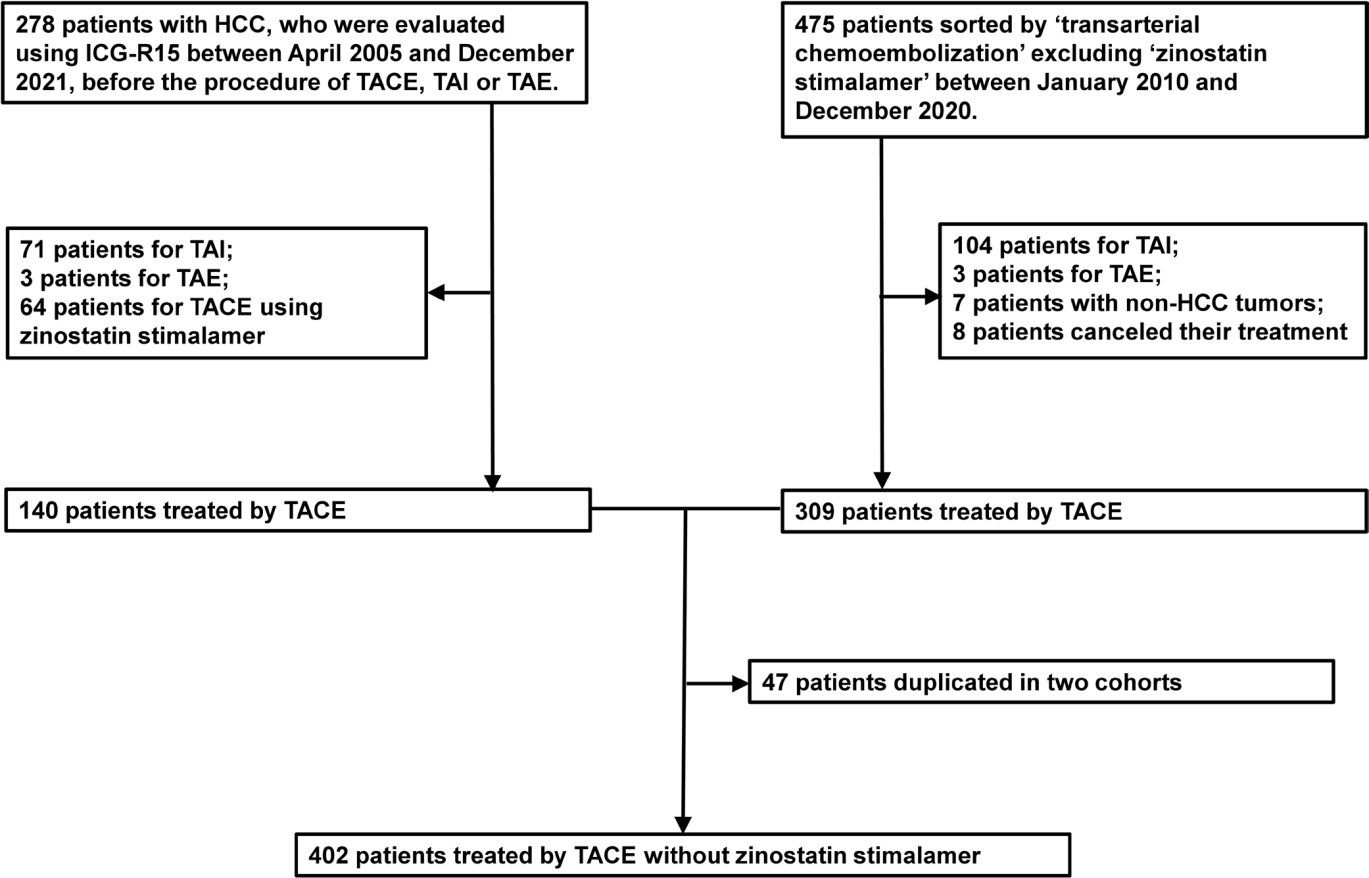
Diagram to select patients for the current cohort. Patients treated by TACE without zinostatin stimalamer were recruited through two arms. The right arm was patients those received indocyanine green retention rate at 15 min before transarterial intervention for hepatocellular carcinoma, as reported in the past study [11] In the left arm, patients were screened with the key word “arterial chemoembolization”, and without “zinostatin stimalamer.” Excluding duplicated data, 402 patients were determined as a study cohort. HCC, hepatocellular carcinoma; ICG‐R15, indocyanine green retention rate at 15 min; TACE, transarterial chemoembolization; TAE: Transcatheter arterial embolization; TAI: Transcatheter arterial infusion.

Patients were recruited from two cohorts (Figure 1). One cohort was identical to the previous study cohort [11], who received indocyanine green retention rate at 15 min before TACE, transcatheter arterial infusion (TAI), or transcatheter arterial embolization (TAE) for HCC [12]. Excluding 71 patients treated with TAI, three patients with TAE, and 64 patients with TACE using zinostatin stimalamer, 140 patients treated by TACE without zinostatin stimalamer were identified in this arm.

In another arm, patients were selected based on the medical records using the keyword “transarterial chemoembolization” and excluding another keyword “zinostatin stimalamer” between 2010 and 2020 (Figure 1). Consequently, 475 patients were identified with a full record of clinical data and imaging at baseline after subtracting the repeated treatment cases for identical patients. Excluding 104 patients treated by TAI, three treated by TAE, seven with malignancies other than HCC, and eight not treated by the transarterial approach, 309 patients treated by TACE without zinostatin stimalamer were identified.

Finally, the duplication of 47 patients was eliminated between the 140‐patient cohort and 309‐patient cohort. Consequently, 402 patients were included in the subsequent analysis.

### 2.4 Indication of Transarterial Chemoembolization

Conventional TACE using an emulsion of ethiodized oil and platinum‐based anticancer drugs was selected for patients with liver function in Child A or B, without bile duct invasion or tumor embolization in the portal vein or its first branches. Drug‐eluting beads TACE has been available since 2014 in cases of tumor diameter (> 5 cm) or the fragile condition of the patient based on performance status or dysfunction of other organs [13, 14]. The indication for TACE was identical in the two cohorts regardless of the year of surgery.

### 2.5 Cardiovascular Thrombosis

Candidate patients with cardiovascular thrombosis were sorted by medication type antiplatelet or anticoagulants including aspirin and other antiplatelet drugs, warfarin and other anticoagulation drugs, ethyl icosapentate and limaprost alfadex. History of arterial or venous thrombotic diseases was confirmed based on the medical records for the sorted patients. Thus, 82 patients were on medication for cardiovascular thrombosis. Among them, three patients were excluded due to medications with limaprost alfadex for spinal canal stenosis or unknown reasons, without any history of cardiovascular diseases. Finally, 79 patients were enrolled as patients with cardiovascular thrombosis (Table 1).

**TABLE 1:** Thrombotic complications and medications.

Cardiovascular thrombotic diseases	Case number
Coronary artery diseases	24
Brain stroke	16
Arrhythmia	15
Portal vein thrombosis	12
Atherosclerosis obliterans	8
Deep venous thrombosis	2
Valvular heart disease	1
Pulmonary embolism	1
Sum	79

Antithrombotic agents	Case number^a^
Aspirin	25
Other antiplatelet agents	21
Warfarin	17
Direct oral anticoagulants	16
Eethyl icosapentate	2
Limaprost Alfadex	2
Sum	83

^a^ Four patients were medicated with two types of drugs.

### 2.6 Clinical Data

Clinical data included age, sex, etiologies of the liver diseases, the date of TACE performance, and its protocol. For the record of blood examination, serum total protein, albumin, aspartate aminotransferase, alanine aminotransferase, total bilirubin (T‐Bil), platelet count, creatinine, prothrombin time (PT), and PT‐INR were considered.

To evaluate tumor burden, the maximum tumor diameter and number of tumor nodules were measured on computed tomography imaging before TACE performance. Staging of tumor progression was designated based on the Clinical and Pathological Study of Primary Liver Cancer [15], The Barcelona Clinic Liver Cancer staging system [16], and The American Joint Committee on Cancer 8th staging system [17].

The functional hepatic reserve was represented by the Child‐Pugh and ALBI scores. ALBI score was calculated according to its original report: Log_10_ T‐Bil (μmol/L) × 0.66 + Albumin (g/L) × (−0.085) [18] T‐Bil (mg/dL) was converted to T‐Bil (μmol/L) according to the equation: T‐Bil (mg/dL) × 17.1.

### 2.7 Statistical Analyses

Continuous variables were presented as median and interquartile range. Mann–Whitney *U* test or Fisher's exact test was applied on analysis of variables. Cox proportional hazard regression analysis was performed to identify independent risk factors for overall survival. Using propensity score matching, the baseline condition was matched between patients without antithrombotic agents and those with thrombotic agents [9]. Especially, the year, when each TACE was performed, was adjusted using the propensity score matching, of equalizing the technical proficiency of operators. Overall survival was calculated using the Kaplan–Meier methods.

JMP Pro 17.0.0 software (SAS Institute Inc., Cary, NC) was adopted to calculate the propensity score. The other analyses were performed using EZR (Saitama Medical Center, Jichi Medical University, Saitama, Japan), a graphical user interface for R software (The R Foundation for Statistical Computing, Vienna, Austria) [19]. Statistical significance was set at *p* < 0.05.

## 3 Results

### 3.1 Baseline Characteristics of Patients

The cohort consisted of 291 males and 111 females with a median age of 72 years and an interquartile range of 64–78 years. Among them, 79 were taking antithrombotic agents for cardiovascular diseases, as shown in Table 1. Consequently, 323 patients without CVT and 79 patients with CVT were identified as the study cohorts, as shown in Table 2.

**TABLE 2:** Baseline characteristics of the cohorts.

	Without CVT	With CVT	*p*
Case number	323	79	—
Background			
Age	73 (66 to 79)	60 (39 to 75)	< 0.0001
Male/female	224/99	67/12	0.0050
Etiologies (HCV/HBV/ETHO/MASH/others)	172/40/51/48/12	32/13/18/14/2	0.0988
Blood examination			
Total protein (g/L)	7.3 (6.8 to 7.7)	7.2 (6.6 to 7.7)	0.3290
Albumin (g/L)	3.7 (3.3 to 4.1)	3.9 (3.4 to 4.1)	0.2832
Platelet count (10^6^/μL)	11.2 (8.0 to 15.7)	12.8 (9.0 to 17.8)	0.0380
Creatinine (mg/dL)	0.75 (0.62 to 0.92)	0.87 (0.73 to 1.05)	0.0002
Total bilirubin (mg/dL)	0.9 (0.6 to 1.3)	0.8 (0.7 to 1.0)	0.4644
AST (U/L)	42 (30 to 61)	35 (25 to 50)	0.0025
ALT (U/L)	29 (20 to 46)	25 (16 to 39)	0.0197
γGTP (U/L)	53 (30 to 94)	57 (32 to 110)	0.4423
Prothrombin time (%)	83 (73 to 95)	80 (60 to 89)	0.0216
PT‐INR	1.10 (1.03 to 1.19)	1.11 (1.06 to 1.29)	0.0657
Functional hepatic reserve			
Child‐Pugh score (5/6/7/8/9/10)	168/102/27/21/3/2	37/22/9/8/2/1	0.1790
ALBI score	−2.433 (−2.785 to −2.021)	−2.594 (−2.820 to −2.161)	0.2537
Tumor burden			
Maximum tumor diameter (mm)	24 (15 to 35)	26 (17 to 40)	0.1896
Number of tumor nodules (1/2/3/4 or more, %)	91/67/49/116 (28/21/15/36%)	31/19/9/20 (39/24/11/25%)	0.0299
JSH stage (1/2/3/4)	32/128/148/15	8/37/32/2	0.2609
AJCC 8th stage (1A/1B/2/3A/3B/4A/4B)	40/45/193/30/4/0/11	9/22/41/5/0/0/2	0.0518*
BCLC stage (0/A/B/C/D)	36/207/64/14/2	5/54/17/2/1	0.6291
TACE			
Protocol			
Ethiodized oil TACE (cisplatin/miriplatin/others)/Beads TACE (epirubicin/others), %	171/106/3/40/3 (53/33/1/12/1%)	27/36/1/12/3 (34/46/1/15/4%)	0.0035
Baseline year (A.D.)	2014 (2011 to 2017)	2016 (2014 to 2018)	< 0.0001
Survival			
Observation period	806 (380 to 1516)	875 (287 to 1519)	0.3523
Overall deaths	169 (52%)	44 (56%)	0.6161
Liver‐related deaths	155 (48%)	33 (42%)	0.3788
HCC‐related deaths	143 (44%)	31 (39%)	0.6456
Deaths not related to liver diseases	14 (4%)	11 (14%)	0.0036
Censored	154 (48%)	35 (44%)	0.6167
Recurrence			
Case number	264 (82%)	61 (77%)	0.3441
Time to recurrence (day)	175 (91 to 388)	238 (133 to 470)	0.0816
Time after recurrence (day)	585 (274 to 1224)	548 (243 to 1234)	0.5076

*Note:* Data were presented as case number or median with interquartile range. *p* < 0.05 was considered statistically significant.

Abbreviations: AJCC, the American Joint Committee on Cancer; ALBI score, albumin bilirubin score; BCLC, The Barcelona Clinic Liver Cancer; CVT, cardiovascular thrombosis; HBV, hepatitis B virus; HCC, hepatocellular carcinoma; HCV, hepatitis C virus; JSH stage, Japan society of Hepatology stage; MASH, metabolic dysfunction‐associated steatohepatitis; TACE, transarterial chemoembolization.

* Stage 3A and 3B, and Stage 4A and 4B were summed up respectively in calculation of Chi‐squared test.

Compared to patients without CVT, those with CVT were significantly younger, had a relatively female‐dominant composition, and had fewer tumor nodules (*p* < 0.05), as shown in Table 2. The functional hepatic reserve and maximum tumor diameter were not significantly different between the two cohorts (*p* > 0.05). For the selection of the TACE protocol, ethiodized oil TACE using cisplatin was adopted more frequently for patients without CVT than for those with CVT. TACE was performed more recently in patients with CVT (*p* < 0.05).

During the observation period, overall and liver‐related deaths did not differ significantly (*p* > 0.05), and deaths due to extrahepatic diseases were significantly higher in patients with CVT complications (*p* < 0.05). Among the 11 patients who died due to extrahepatic diseases, one died of acute myocardial infarction and one died due to complete atrioventricular block. The HCC recurrence rate was not significantly different between the two groups (*p* > 0.05). Time to recurrence and time after recurrence were not significantly different (*p* > 0.05).

### 3.2 The Prognostic Impact of Cardiovascular Diseases in a Crude Analysis

**FIGURE 2:**
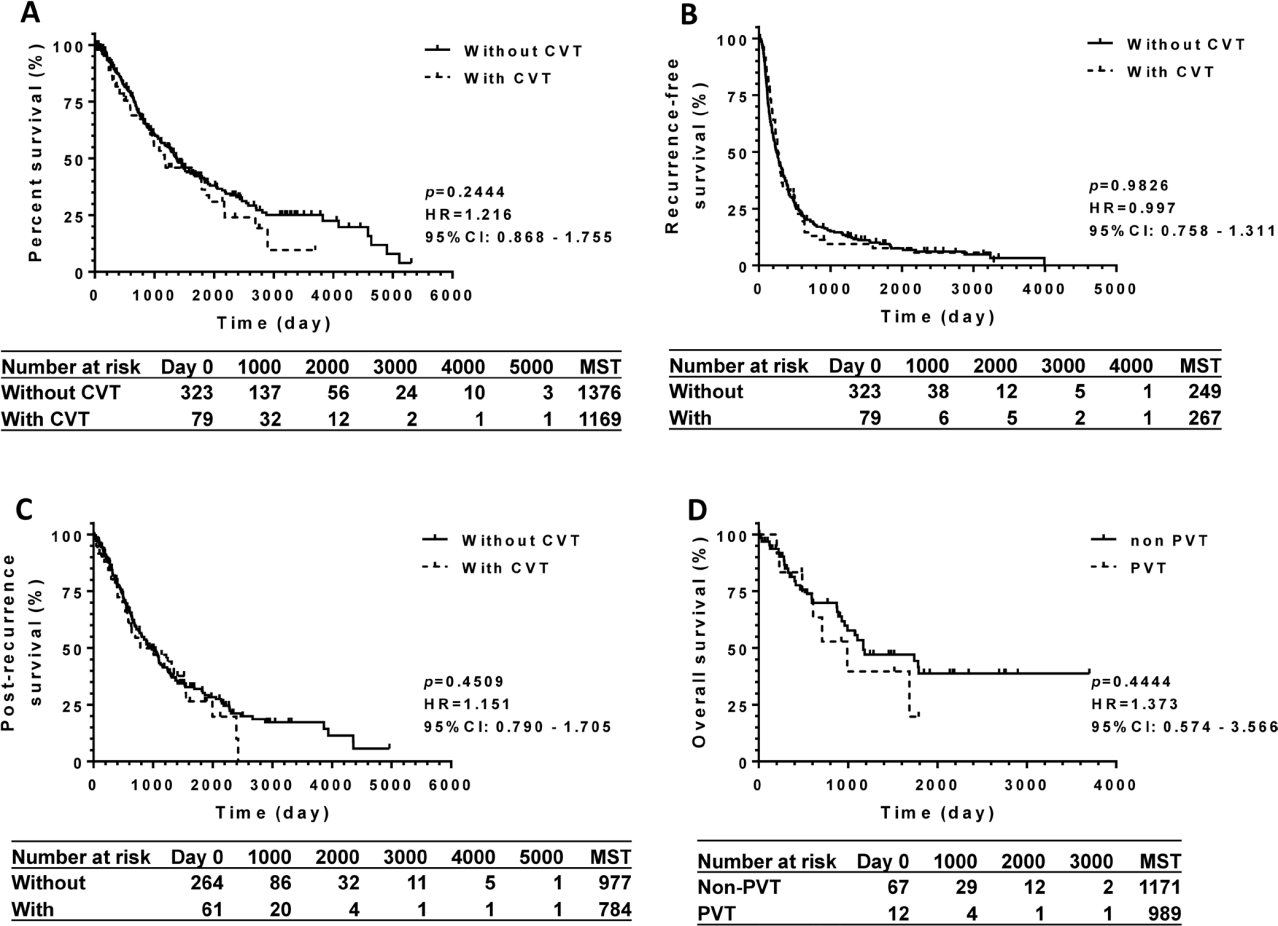
The survival impact of cardiovascular thrombosis on patients with hepatocellular carcinoma. (A) In a crude analysis, the log‐rank test for overall survival presented no significant difference between patients without cardiovascular thrombosis (CVT) and those with CVT (*p* > 0.05). Recurrence‐free survival (B) and post‐recurrence survival (C) were not influenced by CVT complication, either (*p* > 0.05). (D) Overall survival of patients with portal vein thrombosis (PVT) was not significantly differentiated from that of patients with CVTs other than PVT (*p* > 0.05). Number of patients at risk, and median survival time (days) are summarized in the below table. 95% CI, 95% confidence interval; CVT, cardiovascular thrombosis; HR, hazard ratio; MST, the median survival time; PVT, portal vein thrombosis.

To determine the prognostic implications of CVT in patients with HCC after TACE, univariate analysis was performed using a log‐rank test, as shown in Figure 2A. Hence, the overall survival of the 79 patients with CVT was not significantly different from that of the 323 patients without CVT (*p* > 0.05). Recurrence‐free and post‐recurrence survival were not altered by CVT complications (Figure 2B,C). In the CVT group, 12 patients had portal thrombosis (PVT), and 67 had CVTs other than PVT, as shown in Table 1. Overall survival of patients with PVT did not differ from that of patients without PVT (Figure 2D).

Next, multivariate analysis revealed that the complications of CVT significantly worsened the overall survival of patients with HCC after TACE (HR = 1.751 with IQR 1.203–2.548, *p* < 0.05), as shown in Table 3. Age, tumor burden, and ALBI score also impaired the overall survival with statistical significance (*p* < 0.05).

**TABLE 3:** Multivariate analysis of overall survival.

	HR	95% CI	*p*
Age	1.015	1.002 to 1.027	0.0234
Male/female	0.950	0.696 to 1.295	0.7433
Etiology	1.061	0.948 to1.188	0.3026
Maximum diameter	1.011	1.005 to 1.017	0.0005
Number of nodules	1.375	1.220 to 1.551	< 0.0001
Child‐Pugh score	1.147	0.934 to 1.408	0.1900
ALBI score	2.069	1.317 to 3.251	0.0016
TACE Protocol	1.073	0.915 to 1.259	0.3858
Baseline year (A.D.)	0.998	0.952 to 1.046	0.9423
Cardiovascular thrombosis	1.751	1.203 to 2.548	0.0034

Abbreviations: 95% CI, 95% confidence interval; ALBI score, albumin bilirubin score; HR, hazard ratio; TACE, transarterial chemoembolization.

### 3.3 Propensity Score Matching of Baseline Characteristics

To further elucidate the clinical significance of CVT, the baseline characteristics of the two cohorts were matched by propensity score. Age, sex, and etiologies of background liver diseases were selected as variables in terms of demographic heterogeneity; Child‐Pugh and ALBI scores were adjusted to average functional hepatic reserve; and the maximum diameter of tumors and the number of tumor nodules were also included in the calculation of the propensity score to match tumor burden in both cohorts. Thus, a pair of 54 patient cohorts was extracted, as shown in Table 4. Significant differences in platelet count and baseline year at TACE in the original cohort were canceled through matching. The difference in the number of overall death events and the number of censored patients was statistically significant after matching (Table 4). Liver‐ and HCC‐related deaths were not significantly different between the two cohorts.

**TABLE 4:** Baseline characteristics of the matched cohorts.

	Without CVT	With CVT	*p*
Case number	54	54	—
Background			
Age^a^	69 (58 to 74)	72 (57 to 78)	0.4090
Male/female^a^	40/14	45/9	0.3474
Etiologies (HCV/HBV/ETHO/MASH/others)^a^	20/11/9/12/2	24/11/8/9/2	0.3931
Blood examination			
Total protein (g/L)	7.2 (6.9 to 7.7)	7.2 (6.8 to 7.6)	0.6119
Albumin (g/L)	3.9 (3.5 to 4.4)	3.9 (3.4 to 4.1)	0.3709
Platelet count (10^6^/μL)	11.2 (7.1 to 16.2)	12.8 (8.7 to 17.7)	0.0879
Creatinine (mg/dL)	0.70 (0.60 to 0.91)	0.84 (0.69 to 1.01)	0.0062
Total bilirubin (mg/dL)	1.1 (0.6 to 1.4)	0.9 (0.7 to 1.0)	0.2078
AST (U/L)	36 (28 to 52)	35 (25 to 49)	0.3952
ALT (U/L)	24 (19 to 34)	25 (16 to 38)	0.8699
γGTP (U/L)	58 (31 to 118)	56 (31 to 107)	0.8772
Prothrombin time (%)	81 (71 to 94)	83 (63 to 89)	0.5701
PT‐INR	1.13 (1.03 to 1.21)	1.09 (1.06 to 1.25)	0.6917
Functional hepatic reserve			
Child‐Pugh score (5/6/7/8/9/10)^a^	28/18/5/2/0/1	26/15/6/5/1/1	0.4295
ALBI score^a^	−2.626 (−2.830 to −2.161)	−2.556 (−2.820 to −2.156)	0.5827
Tumor burden			
Maximum tumor diameter (mm)^a^	23 (15 to 36)	23 (16 to 39)	0.7259
Number of tumor nodules (1/2/3/4 or more, %)^a^	25/9/6/14 (46/17/11/26%)	22/12/5/15 (41/22/9/28%)	0.7000
JSH stage (1/2/3/4)	9/17/21/7	7/25/20/2	0.3015
AJCC 8th (1A/1B/2/3A/3B/4A/4B)	12/8/24/3/2/0/5	8/14/27/3/0/0/2	0.3547*
BCLC stage (0/A/B/C/D)	9/17/21/7	7/25/20/2	0.2056
TACE			
Protocol			
Ethiodized oil TACE (cisplatin/miriplatin/others)/Beads TACE (epirubicin/others), %	32/15/0/7/0 (59/28/0/13/0%)	18/27/1/5/3 (33/50/2/9/6%)	0.0174
Baseline year (A.D.)	2016 (2012 to 2018)	2015 (2014 to 2017)	0.6568
Survival			
Observation period	1190 (446 to 2413)	740 (288 to 1790)	0.1679
Overall deaths	20 (37%)	33 (61%)	0.0205
Liver‐related deaths	20 (37%)	24 (44%)	0.5571
HCC‐related deaths	17 (31%)	23 (43%)	0.4621
Deaths not related to liver diseases	0 (0%)	9 (17%)	< 0.0001
Recurrence of HCC	44 (81%)	41 (76%)	0.6390
Censored	34 (63%)	21 (39%)	0.0205
Recurrence			
Case number	44 (81%)	41 (76%)	0.6390
Time to recurrence (day)	207 (123 to 472)	232 (140 to 382)	0.9633
Time after recurrence (day)	848 (289 to 1983)	566 (193 to 1434)	0.2245

*Note:* Data were presented as case number or median with interquartile range. *p* < 0.05 was considered statistically significant.

Abbreviations: ALBI score, albumin bilirubin score; CVT, cardiovascular thrombosis; HBV, hepatitis B virus; HCC, hepatocellular carcinoma; HCV, hepatitis C virus; JSH stage, Japan society of Hepatology stage; MASH, metabolic dysfunction‐associated steatohepatitis; TACE, transarterial chemoembolization.

^a^ Variables were adjusted using a propensity score‐matched analysis.

* Stage 3A and 3B, and Stage 4A and 4B were summed up respectively in the calculation of the Chi‐squared test.

### 3.4 Log‐Rank Analysis for Prognoses in the Two Matched Cohorts

**FIGURE 3:**
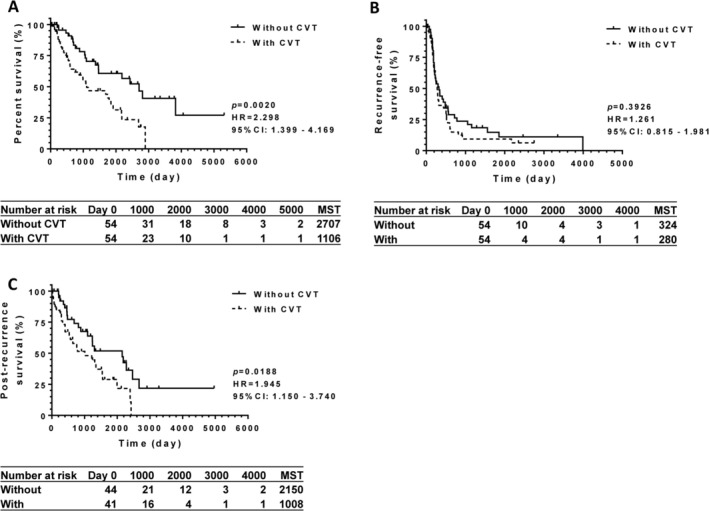
The matched pair of cohorts was generated based on propensity score. After adjustment, (A) the overall survival of patients with cardiovascular thrombosis (CVT) significantly shortened to half compared to those not complicated with CVT (*p* < 0.05). (B) The recurrence‐free survival in the matched pair of 54‐patient cohorts was not significantly different compared to those not complicated with CVT (*p* > 0.05). (C) Post‐recurrence survival was compared between 44 patients without CVT and 41 patients with CVT. Post‐recurrence survival was two times longer in patients without CVT, compared to those with CVT with statistical significance (*p* < 0.05). Number of patients at risk, and median survival time (days) are summarized in the below table. 95% CI, 95% confidence interval; CVT, cardiovascular thrombosis; HR, hazard ratio; MST, the median survival time.

The difference in the overall survival between the two matched cohorts was determined using the log‐rank test. As shown in Figure 3A, the median survival time of patients with CVT was half in length compared to that of those without CVT (*p* < 0.05).

Tumor recurrence after TACE was detected in 44 patients without CVT and 41 patients with CVT(Table 4). Recurrence‐free survival was not significantly different between the two matched cohorts (*p* > 0.05; Figure 3B). The median recurrence‐free survival time was < 1 year in both cohorts. Post‐recurrence survival was significantly extended in the group without CVT compared to the group with CVT (*p* < 0.05), as shown in Figure 3C.

## 4 Discussion

This single‐center study determined the prognostic impact of CVT in patients with HCC treated with TACE. Crude and adjusted analyses revealed that CVT shortened the overall survival by up to half compared to those without CVT. Shorter overall survival is attributed to shorter post‐recurrence survival. After adjusting for background differences, CVT complications resulted in more frequent deaths that were unrelated to liver disease.

Accumulating evidence has revealed a bidirectional causal relationship between CVT and cancer progression. Cancer‐associated arterial thrombotic events are increasingly being recognized, particularly in specific malignancies and in association with specific anticancer therapies [20]. Therapeutic intervention, including radiotherapy, often induces arterial thrombosis through mechanisms involving endothelial injury [21].

Cancer‐associated venous thrombosis may be more common than arterial thrombosis [22]. Patients with cancer have up to four times higher risk of venous thromboembolism than the general population, which is associated with significant morbidity and mortality [23]. Because having cancer poses a persistent and progressive risk for venous thrombosis, its treatment is generally indefinite [24].

In addition to prior malignancy‐based thrombus formation, thrombus‐promoting factors inversely promote cancer progression. Platelets have the potential to promote cancer development through various bioactive molecules [25]. In the tumor microenvironment, platelets stimulate fibrosis and tumorigenesis by secreting pro‐fibrogenic factors, interacting with monocytes and macrophages, and regulating the innate immune response [26]. Experimentally induced thrombocytopenia has antimetastatic effects in a mouse model [27].

Similar to platelets, coagulation factors enhance tumor growth. Upstream and downstream factors in the coagulation cascade may be involved in different stages of cancer, including tumorigenesis, primary tumor growth, and metastasis [28]. A prime example is tissue factor, which is the initial protein of the coagulation cascade. Tissue factor/VII promotes tumor growth by activating protease‐activated receptor 2 signaling and increasing the expression of angiogenic factors such as vascular endothelial growth factor [29].

Based on the premise that coagulation stimulates tumor development at several stages, the effectiveness of anticoagulation therapy has been discussed as a strategy for cancer treatment. Among antiplatelet agents, aspirin (acetylsalicylic acid) is recognized as a promising cancer‐preventive agent. Daily administration of low‐dose aspirin is recommended by the US Preventive Services Task Force as a primary preventive measure for colorectal cancer [30]. A recent meta‐analysis found that aspirin use is associated with a decreased risk of hepatocellular carcinoma [31]. Another study supported the use of aspirin to reduce the incidence of hepatocellular carcinoma and liver‐related mortality in at‐risk individuals. Hepatocellular carcinoma recurrence after treatment is lower in patients receiving NSAIDs [32]. Among anticoagulants, edoxaban induced apoptosis in a mouse colon cancer cell line and significantly inhibited colon cancer tumor cell growth [33].

In summary, (1) CVT and cancer are causally related, and (2) antithrombotic agents used in CVT treatment potentially suppress tumor progression. Therefore, the prognostic impact of CVT is assumed to negatively modulate the overall survival, and antithrombotic therapy is expected to inhibit the formation of the tumor microenvironment.

Our results revealed that the overall and post‐recurrence survival rates of patients with CVT were significantly reduced by half (Figure 3A). HCC‐related mortality was not changed, as shown in Tables 2 and 3. In addition, deaths unrelated to liver disease increased in patients with CVT (Tables 2 and 3). Recurrence‐free survival was not improved by antithrombotic agents, as shown in Figure 3B.

These results might be attributed to the fact that antithrombotic agents were not sufficiently effective in improving HCC‐related mortality, overall survival, and recurrence‐free survival in patients with HCC subject to TACE. As suggested by the significant difference in post‐recurrence survival, coexisting CVT may lead to more frequent deaths unrelated to HCC in patients with CVT. Therefore, CVT might be considered an indicator of functional cardiovascular reserve in patients with HCC, as Child‐Pugh classification and the ALBI score are indices of functional hepatic reserve. Following functional hepatic reserve and tumor burden, CVT may be a major prognostic factor in patients with HCC. It may be possible to make the expected survival time in patients with CVT. Based on the shorter post‐recurrence survival of patients with CVT, more curative methods should be selected for such patients.

The findings of this study are applicable to a large proportion of patients with HCC. Based on the Japanese medical claims database, the most common therapeutic choice for HCC is TACE [34]. Further analysis should be performed in patients with absolutely resectable HCC at an early stage, or in patients with no indication for TACE due to macrovascular invasion or extrahepatic metastasis of the tumor.

Despite an overall decline in the burden of primary liver cancer in the Asia–Pacific region over the past decade, an increase in its incidence has been noted for several etiologies, including MASLD and ALD. However, viral hepatitis remains the leading cause of death, accounting for > 60% of the total burden [35]. MASLD and ALD are independent risk factors for CVT and increase the susceptibility to hypertension, atherosclerosis, arrhythmia, myocardial dysfunction, cardiac valve deformation, and venous thrombosis through putative mechanisms, including systemic inflammation, endothelial dysfunction, oxidative stress, insulin resistance, and altered lipid metabolism [36].

In terms of post‐TACE therapeutic strategies, an emerging trend in systemic therapy is being carefully applied in patients with CVTs. Recently, angiogenesis inhibitors have been adopted as the first or second choice of treatment for unresectable HCC in therapeutic guidelines [37, 38, 39]. Bevacizumab, an anti‐vascular endothelial growth factor (VEGF) human monoclonal antibody, is positioned as first‐line treatment with atezolizumab, an immune checkpoint inhibitor [40]. Bevacizumab can potentially increase the prevalence of major adverse cardiovascular events and venous thrombosis [41, 42]. The second‐line choice of therapy includes a series of multikinase inhibitors: sorafenib, lenvatinib, regorafenib, and ramucirumab. Sorafenib potentially increases the incidence of cardiovascular events in patients with HCC [43].

In the current study, TACE using zinostatin stimalamer was not evaluated. Zinostatin stimalamer is a chemical conjugate of a synthetic copolymer of styrene‐maleic acid and an anticancer antibiotic protein neocarzinostatin [44]. Arterial injection of zinostatin stimalamer sometimes causes severe complications such as hepatic arterial obstruction, liver abscess, and hepatic failure [45]. After the safety profile of miriplatin was proved superior to zinostatin stimalamer, the latter became no longer used for TACE [46]. Therefore, data of TACE using zinostatin stimalamer were excluded in the evaluation above.

This study has some limitations. First, this retrospective study lacked data validation. Under the given conditions, the patient data were accumulated as the maximum and longest possible. Second, patients with CVT may not be identical to those treated with anticoagulants. While maximum effort was made to exclude confounding factors using propensity score matching, the potential number of patients in the group without CVT might have been complicated by CVT. Third, it was unclear that TACE treatment at baseline was the initial treatment for each patient. A certain number of patients might be treated in advance through surgery, RFA, or TACE using zinostatin stimalamer.

## 5 Conclusion

CVT significantly impaired the overall survival and post‐recurrence survival in patients with HCC treated with TACE. CVT may be a major prognostic factor in patients with HCC, after tumor burden and functional hepatic reserve. Assuming that the life expectancy of patients with CVT is only half that of patients without CVT, the therapeutic strategy should be adjusted in patients with CVT.

## Author Contributions

**Koji Fujita:** conceptualization, methodology, data curation, formal analysis, writing. **Kei Takuma:** data curation. **Mai Nakahara:** data curation. **Hironobu Suto:** data curation. **Asahiro Morishita:** formal analysis. **Takashi Himoto:** validation. **Keiichi Okano:** validation. **Hideki Kobara:** supervision.

## Funding

The authors have nothing to report.

## Ethics Statement

This study protocol was reviewed and approved by the Institutional Review Board of Kagawa University, Faculty of Medicine (Serial number: 2024‐178).

## Consent

The requirement for informed consent was waived because the clinical data were freely accessible. We provided opt‐out methods for the relatives of the dead participants by publishing a summary of this study on our university website.

## Conflicts of Interest

The authors declare no conflicts of interest.

## Data Availability

The data that support the findings of this study are available from the corresponding author upon reasonable request.
